# Acute Wound Healing Potential of Marine Worm, *Diopatra claparedii* Grube, 1878 Aqueous Extract on Sprague Dawley Rats

**DOI:** 10.1155/2020/6688084

**Published:** 2020-12-28

**Authors:** Nor ‘Awatif Che Soh, Hannah Syahirah Rapi, Nurul Shahirah Mohd Azam, Ramesh Kumar Santhanam, Suvik Assaw, Mohd Nizam Haron, Abdul Manaf Ali, M. Maulidiani, Izwandy Idris, Wan Iryani Wan Ismail

**Affiliations:** ^1^Cell Signaling and Biotechnology Research Group (CeSBTech), Faculty of Science and Marine Environment, Universiti Malaysia Terengganu, 21030 Kuala Nerus, Terengganu, Malaysia; ^2^Biological Security and Sustainability (BioSeS) Research Group, Faculty of Science and Marine Environment, Universiti Malaysia Terengganu, 21030 Kuala Nerus, Terengganu, Malaysia; ^3^Faculty of Science and Marine Environment, Universiti Malaysia Terengganu, 21030 Kuala Nerus, Terengganu, Malaysia; ^4^School of Animal Science, Faculty of Bioresources and Food Industry, Universiti Sultan Zainal Abidin, 22200 Besut, Terengganu, Malaysia; ^5^School of Agriculture Science and Biotechnology, Faculty of Bioresources and Food Industry, Universiti Sultan Zainal Abidin, 22200 Besut, Terengganu, Malaysia; ^6^South China Sea Repository and Reference Centre, Institute of Oceanography and Environment (INOS), Universiti Malaysia Terengganu, Kuala Terengganu, Terengganu, Malaysia

## Abstract

*Diopatra claparedii* which is colloquially known as Ruat Sarung can be found along the west coast of Peninsular Malaysia. The species has a unique ability to regenerate anterior and posterior segments upon self-amputation or injury, thus having potential as a wound healing promoter. In this study, the wound healing potential of *D. claparedii* aqueous extract on acute wound model in rats was revealed for the first time. Various concentrations (0.1%, 0.5%, and 1.0% w/w) of *D. claparedii* ointment were formulated and tested on Sprague Dawley rats through topical application on full-thickness skin wounds for 14 days. The wound healing effects were investigated via behaviour observation, wound contraction, and histopathological analysis. Quality assessment was performed via skin irritation test, microbial contamination test (MCT), and heavy metal detection. The study also included test for antibacterial activities and detection of bioactive compounds in *D. claparedii*. One percent of *D. claparedii* ointment showed rapid wound healing potential with good soothing effects and more collagen deposition in comparison to the commercial wound healing ointments such as acriflavine (0.1% w/v) and traditional ointment gamat (sea cucumber extract) (15.0% w/v). No local skin irritation, microbial contamination, and insignificant concentration of heavy metals were observed, which indicate its safe application. Moreover, the aqueous extract of *D. claparedii* exhibited antibacterial activities against *Escherichia coli* and *Pseudomonas aeruginosa* with minimum inhibitory concentration (MIC) value at 0.4 g/ml. ^1^H NMR analysis of the aqueous extract of *D. claparedii* revealed some metabolites that might be responsible for its wound healing properties such as amino acids, halogenated aromatics, organic acids, vitamins, and others. Altogether, these results suggested that the aqueous extract of *D. claparedii* could be utilised as an alternative natural wound healing promoter.

## 1. Introduction

Skin is the human body's largest organ, representing 15% of total adult body weight, and acting as mechanical barriers to external environment. Any skin injury allows foreign materials to invade the host body [1]. Wound, by definition, is breaks in skin epithelial integrity and may cause further disruption in skin anatomy, physiology, and its functions. There are two types of wound known as acute and chronic. Acute or minor wound is an everyday global public health problem. Physiologically, an acute wound takes four to six weeks to heal completely. However, if the healing process has prolonged more than six weeks, without any sign of resolution, it will lead to chronic or more severe wound [1, 2]. Chronic wound will cause a huge burden on a patient especially due to high-cost treatment and many health complications such as gangrene that can lead to amputations in diabetic patients [3]. This incidence can be avoided if more attention is given to treat acute wound effectively, with higher priority, and is the major focus in this study.

Wound healing is a complex process involving four phases which are haemostasis, inflammation, proliferation, and remodelling phase [4]. A proper wound management including effective treatment is essential to achieve complete wound healing process that would halt progression of acute wound to chronic wound. Recently, many researchers are seeking natural products with superior healing power associated with minimal adverse effects to target the wounds due to limitations with current treatment. It is believed that these efforts could manage the inline wound care issues such as inadequate supply of drugs, drug resistance, side effects from synthetic drugs, and economic burden [5]. Among natural products used in wound healing treatment are honey and sea cucumber species, *Stichopus herrmanni* [6]. Honey is known to have many benefits for human health including wound healing. However, its composition is difficult to standardize due to variations in honey types. Also, the existence of fake honey is a challenge [6]. Sea cucumber locally known as gamat is a marine organism from class Holothuroidea that has an effective wound healing agent due its antioxidant, antibacterial, and anti-inflammatory properties [7]. However, current practice of harvesting gamat from wild for extraction is not sustainable [6]. Thus, polychaetes with high abundance and diversity can be explored for their potential as an alternative wound healing agent.

Polychaete is a marine worm that belongs to phylum Annelida. It is a small invertebrate that inhabits all places in a marine environment and contributes to a high percentage of the total macrobenthic community diversity, abundance, and biomass [8, 9]. In Malaysia, 64 species of polychaete that come from 31 families have been recorded from 1866 to 2013 [10]. Polychaetes have the ability to regenerate themselves upon injury or self-amputation. The ability is paralleled to the polychaete's natural habitat, in which it is exposed to predatory attacks such as birds and fish targeting the anterior or posterior part of the body. The ecological stresses have led the polychaete into the self-regeneration capability and evolution for adaptation and sustainable life to its natural habitat. This regeneration ability is different from species to species [11].

*Diopatra claparedii* locally known as Ruat Sarung is a local marine worm. This species is found widely at mudflat in mangrove or estuary area along the west coast of Peninsular Malaysia [10]. It has a segmented body with parapodia that bear chaetae. This genus lives in self-made tubes that project from the sediment on the seabed [11]. Thus, they can be easily found by bait diggers when low tides and sold for fishing purposes. Moreover, it has a unique feature where it can regenerate both anterior and posterior segment upon injury or self-amputation [12]. Due to this distinct feature, it has been hypothesised to have wound healing potential, yet no reports were available until today. In phylum Annelida, many polychaetes were reported to possess significant medical applications such as anti-cancer, anti-inflammatory, and antimicrobial agents, but there is still less information about polychaete application in wound healing treatment [13]. Therefore, the study intends to discover the wound healing potential and chemical properties of the *D. claparedii* aqueous extract using full-thickness wound in animal model.

## 2. Materials and Methods

### 2.1. Reagents

Diethyl ether, methyl blue, nutrient broth, phosphomolybdic acid, and Harris and Weigert's iron hematoxylin were purchased from Merck, USA. Gentamycin, and picric acid moistened with water, ≥98%, were obtained from Sigma-Aldrich, USA. Acid fuschin was acquired from R & M, Malaysia. Meanwhile, bacteriological agar and Mueller-Hinton broth (MHB) were acquired from Oxoid, UK.

### 2.2. Preparation of *Diopatra claparedii* Aqueous Extract and Ointment

Fresh specimens of *D. claparedii* polychaete species were collected from the west coast of peninsular Malaysia. Polychaete was removed from the tube using scissors and rinsed with distilled water. Then, the sample was stored in the −80°C freezer. The method was conducted according to Mazliadiyana et al. and Rapi et al. with some modifications [7, 14]. The sample was thawed at room temperature and 51.58 g of sample was weighed prior to being cut into small pieces and pulverised using pestle and mortar. Then, approximately 515 ml of distilled water was added to the samples with volume ratio was 1 : 10 and soaked overnight. The sample was filtered using Whatman filter paper no. 1 and collected in a flask (Flask A). Next, the residue from the filtration was soaked again in distilled water for 4 h and centrifuged at 3000 rpm for 20 min. Later, the supernatant was collected and placed in another flask (flask B). Finally, the solutions in flasks A and B were mixed and stored at −80°C. After 24 h, the samples were freeze-dried into a powder form. The powder extract of *D. claparedii* was weighed and mixed into cetomacrogol emulsifying ointment at various concentrations (0.1%, 0.5% and 1.0% w/w). The mixture was mixed well using sterile mortar and pestle until the homogenised ointment was obtained.

### 2.3. Animals and Study Design

The protocol of the study was approved by the Universiti Sultan Zainal Abidin (UniSZA) Animal and Plant Research Ethics Committee (UAPREC) (Ref no. UAPREC/04/040). A total of 35 female Sprague Dawley rats weighed between 130 and 180 g and age between 8 and 10 weeks were obtained from UniSZA. Female rats are used for the study due to their availability. Prior to the experiment, the animals were housed individually in cages under a 12 h light and 12 h dark cycle in a temperature-controlled room (22 ± 3°C) for a week to adapt to the laboratory conditions. They were allowed free access to standard laboratory animal diet with water *ad libitum*. Then, the animals were randomly (*n* = 5) assigned to seven groups: three different concentrations of polychaete ointment (PO) (0.1%, 0.5%, and 1.0% w/w), two positive groups (15.0% gamat oil and 0.1% acriflavine), and two negative controls; the rats were treated with cetomacrogol emulsifying ointment only and also untreated group wherein no treatment was applied.

### 2.4. *In Vivo* Wound Model

The method for wound model was carried out according to Rapi et al. [14] and Dunn et al. [15] with slight modifications. Prior to surgery, the shaved skin was disinfected with 10% of povidone-iodine. Then, a pair of full-thickness circular wounds about 8 mm in diameter were created on dorsum of the rats using disposable biopsy punch after anaesthetised by 100% of diethyl ether. Wounds were treated topically once daily for 14 days according to the above-mentioned groups. About 30 to 40 mg of ointment was applied topically on the wound. The wound area was measured on days 0, 3, 7, 11, and 14 after the trauma by tracing the wound margin using a transparent film and a graph paper. The wound healing percentage (WHP) was calculated using the following formula [16]:

(1) $$\begin{array}{c} \text{WHP}=\frac{\left[\left(\text{wound area day 0}\right)-\left(\text{wound area on day }X\right)\right]}{\text{wound area day 0}}\times 100, \end{array}$$

*X* = days 3, 7, 11, and 14 of post-trauma.

### 2.5. Behaviour Observation

The behaviour of each rat was assessed according to Hashim et al. [17] and Vukojevic et al. [18] with a slight modification. The behaviour was observed and recorded if there was presence of any withdrawal response to pain such as action of licking or scratching at wound area. Besides, the presence of other activities such as sleeping, resting, and cage exploration (walking and climbing) was also recorded. The observation was done in the morning for 30 min while handling the experiment on days 0, 3, 7, 11, and 14.

### 2.6. Gross Observation

The presence of granulation tissues, cardinal signs of inflammation (erythema and oedema), scab, epithelial tissue, and growth of fur was observed on days 0, 3, 7, and 14 following treatments [16].

### 2.7. Histology Observation

All rats from each group were sacrificed on day 14. The skin specimen was harvested with a 2 mm border of unwounded skin tissue. The tissue samples were fixed in 10% neutral buffered formalin, processed, and embedded in paraffin wax prior to being sectioned into 5 *μ*m thin tissue ribbon. The prepared slides were stained with hematoxylin and eosin (H & E) for general histology observation and Masson's trichrome (MT) for collagen deposition and arrangement of wounded tissue [19].

### 2.8. Quality Assessment of Aqueous Extract Emulsifying Ointment of *D. claparedii*

#### 2.8.1. Skin Irritation Test

Safety of the samples (PO: 0.1%, 0.5%, and 1.0% w/w) and cetomacrogol ointment were observed by conducting skin irritation study as per the Organisation for Economic Co-operation and Development (OECD) guideline, number 404 [20]. The total irritation score including presence of oedema and erythema was calculated.

#### 2.8.2. Microbial Contamination Test (MCT)

The best concentration of PO from *in vivo* assessment on wound healing was chosen to test for microbial contamination test (MCT), which was 1.0% of PO. The procedure was adapted from British Pharmacopoeia [21]. MCT reveals the total aerobic microbial count (TAMC), total yeast and mould count (TYMC), and the presence of microbes such as *Staphylococcus aureus* and *Pseudomonas aeruginosa*. To test for TAMC and TYMC, 1 g of ointment was dispersed in Ringer solution containing 0.25% of tween 80. Then, the mixture was diluted to 1:10 of dilution. About 1 ml of mixture was spread onto tryptone soy agar (TSA) and Sabouraud dextrose agar (SDA) to test for TAMC and TYMC accordingly. TSA was incubated at 30–35°C for three to five days and SDA was incubated at 20–25°C for five to seven days. Colonies that grow onto plates were calculated and expressed in CFU/g. To test the presence of *S. aureus* and *P. aeruginosa*, 10 g of ointment was dissolved in 90 ml of buffered sodium chloride (NaCl) peptone solution. Then, 10 ml of mixture was added to 90 ml of tryptone soy broth (TSB) and incubated at 30 to 35°C for 18 to 24 h. After incubation, the mixture was sub-cultured onto mannitol salt agar (MSA) and cetrimide agar (CETA) to test for *S. aureus* and *P. aeruginosa* accordingly. MSA and CETA were incubated at 30–35°C for 18 to 72 h. The growth of *S. aureus* and *P. aeruginosa* was observed and recorded.

#### 2.8.3. Heavy Metals Detection

Heavy metals composition in PO that includes arsenic, cadmium, lead, and mercury was analysed by My CO2 Laboratory, Shah Alam, Selangor, Malaysia, using the Association of Official Agricultural Chemists (AOAC) method [22]. The best concentration of PO from *in vivo* assessment on wound healing was chosen to test the heavy metals composition which was 1.0% of PO.

### 2.9. Antibacterial Activities

Antimicrobial activity was evaluated using minimum inhibitory concentration (MIC) assay via microdilution method and minimal bactericidal concentration (MBC) assay based on Clinical Laboratory Standards Institute [23]. Five different species of bacteria such as *S. aureus* ATCC 25923, *S. epidermidis* ATCC 14990*, Escherichia coli* ATCC 25922, *P. aeruginosa* ATCC 27853, and *Klebsiella pneumonia* ATCC 700603 were selected for the test. Initially, 100 *µ*l of stock solution of polychaete extract (0.4 g/ml) was dissolved in sterile distilled water and loaded in the first row of 96-well plate. Then, it was followed by twofold dilutions of polychaete extract in subsequent rows that previously dispensed with 50 *µ*l of Mueller-Hinton broth (MHB). The last row was the control only with MHB, to confirm the bacterial growth in MHB in the absence of antibacterial agent. Then, 50 *µ*l of bacterial inoculums (1.5 × 10^6^ CFU/mL) was inoculated in each well. Direct suspension colony method was used to prepare bacterial inoculum. Then, the 96-well plate was incubated at 37°C for 18 h.

The MIC is the lowest concentration of extract that completely inhibits growth of the bacteria in 96-well plate. To confirm the MIC endpoint, about 30 *µ*l of 5 mg/ml tetrazolium salts, 3-(4,5-dimethylthiazol-2-yl)-2,5-diphenyltetrazolium bromide (MTT), was added to each well and incubated for 1 h. Colour changes from yellow to purple represent that the presence of bacteria reduced the MTT into formazan. No colour change represents the absence of bacterial growth. All the tests were done in triplicate. For MBC assay, sub-cultures were done in Mueller-Hinton agar (MHA) from the wells that displayed the absence of bacterial growth in MIC assay. Plates were incubated for 18 h at 37°C and the growth of bacteria was analysed.

### 2.10. Sample Preparation for Proton Nuclear Magnetic Resonance (^1^H NMR)

For ^1^H NMR experiment, about 10 mg of freeze-dried *D. claparedii* extract was transferred into a clean vial. Then, 1 ml of deuterated dimethyl sulfoxide (DMSO-d_6_) containing 0.03% (v/v) tetramethylsilane (TMS) was added and the mixture was vortexed until the extract was dissolved completely. The mixture was centrifuged at 13,000 rpm for 10 minutes and the supernatant was transferred to an NMR tube *prior* to ^1^H NMR analysis. ^1^H NMR experiment was carried out on a 400 MHz (Bruker Avance 400 MHz, Germany) using the following parameters: temperature 26°C, pulse width (PW) 21.0 *µ*s (90°), and relaxation delay (RD) 2.0 s and acquisition time 4.29 min (64 scans). A standard water-suppressed one-dimensional NMR was applied using the PRESAT sequence in order to remove the residual of water signal at 3.30 ppm. The spectral pre-processing that includes phasing and baseline corrections was conducted using evaluation version Chenomx Processor software (version 7.62, Alberta, Canada). The metabolites were identified based on their ^1^H NMR characteristics and comparison with the NMR spectra of reference compounds available via Chenomx Profiler software (version 7.62, Alberta, Canada), online database (https://hmdb.ca/), and published literature.

### 2.11. Statistical Analysis

The values of WHP were expressed as mean ± SEM and analysed using SPSS version 22.0. One-way analysis of variance (ANOVA) followed by Tukey's HSD post hoc test was used to analyse and compare the mean values [7]. The differences between mean values were considered significant when *p* < 0.05. The graphical representation was done using Microsoft Excel 2010.

## 3. Results

### 3.1. Animal Behaviour

The behaviour of Sprague Dawley rats towards the treatment was observed after the trauma (day 0) was inflicted and continued as wound healing progressed on days 3, 7, 11, and 14. Three criteria were monitored including withdrawal pain response (wound licking/scratching), sleeping/resting, and cage exploration (walking/climbing). Results from the observation demonstrated that rats from all groups showed a similar pattern of behaviour throughout the experimental period (Table 1). For instance, on day 0, all rats showed strong withdrawal responses to pain after completing procedure of excisional wound. They tried to lick or scratch at the wound area. On day 3, all rats were at inactive mode by showing sleeping or resting activity. Meanwhile, on day 7, they started to walk around inside the cage and became more active and started to climb around the cage on days 11 and 14.

### 3.2. Gross Observation and Wound Contraction

**Figure 1:**
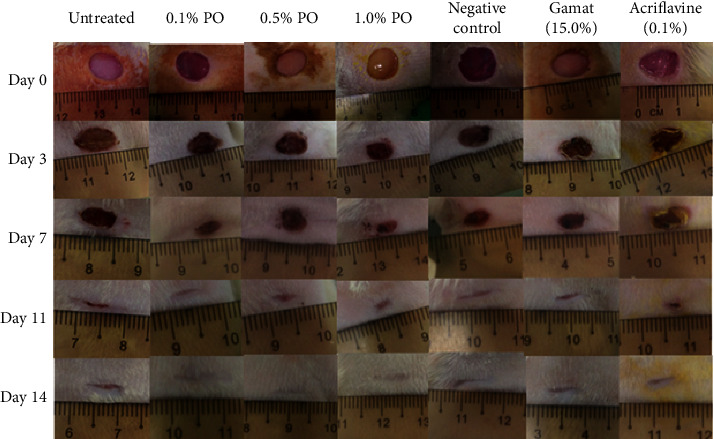
Gross observations of wound healing progression of 8 mm in diameter of excisional wound on dorsum of Sprague Dawley rat. Seven treatment groups were tested which were 0.1%, 0.5%, and 1.0% of polychaete ointment (PO), gamat (15%), acriflavine (0.1%), cetomacrogol emulsifying ointment (negative control), and untreated group. The observation showed the wound was healed gradually within 14 days of experimental period (*n* = 5).

Basically, wound observation was conducted visually to report the health status of wound condition such as no sign of infection or pus (Figure 1). At day 0, all the wound areas on the rats' body were found slightly bleeding after completing the wound procedure and it took a few minutes to coagulate. On day 3, all the wound surfaces demonstrated the presence of blood clot and decreased presence of oedema as a late sign of inflammation during the second phase of wound healing (inflammation phase). Granulation tissue started to appear with reddish in colour for all groups except the rats treated with acriflavine, where the wound area appeared blackish in colour on day 3 due to the colour staining of the acriflavine (yellow lotion) on the wound surface which results in darkening of scab. On day 7, there were obvious wound contractions with dry scab observed in all the groups especially the wound treated with 1.0% of PO. On day 11, complete re-epithelialisation was seen in almost all the groups except for rats from the untreated and acriflavine treated group. On day 14, all the treated wounds appeared fully closed except for untreated group. Moreover, wound treated with PO showed earlier remodelling phase which manifested with the appearance of fur around the wounded tissues.

**Figure 2:**
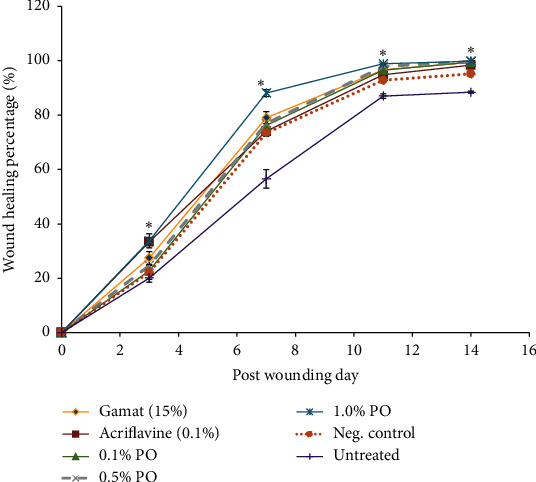
Wound healing percentage of different treatment groups measured on days 3, 7, 11, and 14 after wounding. The wound contraction was measured as percentage reduction from original wound size. Seven treatment groups were tested which were 0.1%, 0.5%, and 1.0% of polychaete ointment (PO), gamat (15%), acriflavine (0.1%), cetomacrogol emulsifying ointment (negative control), and untreated group. The result showed that wound treated with 1.0% PO demonstrated rapid wound closure as compared to other treatment groups. Data are mean ± SEM (*n* = 5). The significance difference was analysed using one-way ANOVA. ^*∗*^*p* < 0.05 was compared between 1.0% PO, negative control, and untreated group.

In this study, wound contraction was evaluated by calculating the wound healing percentage (WHP). The wound closure was measured on days 3, 7, 11, and 14 as compared to original wound size on day 0 (Figure 2). In general, sizes of all the designated wounds were reduced gradually since day 0 up to day 14. For wounds treated with PO, they were healed in accordance to concentration-dependent of the PO with 1.0% of PO showing the highest WHP. Moreover, the same concentration of PO showed the fastest wound healing rate throughout 14 days of experimental period compared to other groups including positive control. Besides, 1.0% of PO showed the highest wound healing rate (99.8 ± 0.1%) and untreated group demonstrated the lowest wound healing rate (88.4 ± 0.4%) on day 14. Furthermore, 1.0% of PO significantly (*p* < 0.05) healed the wound faster compared to rats treated with 0.1% acriflavine on days 7 and 11. Wound healing percentage of 1.0% of PO was 88.1 ± 1.3% and 98.9 ± 0.3% on days 7 and 11, respectively. Meanwhile, acriflavine showed WHP of 74.1 ± 2.3% on day 7 and 94.89 ± 1.11% on day 11. Interestingly, there was no significant difference in WHP between 1.0% PO and 15% gamat oil throughout the treatment course.

### 3.3. Histology Analysis

**Figure 3:**
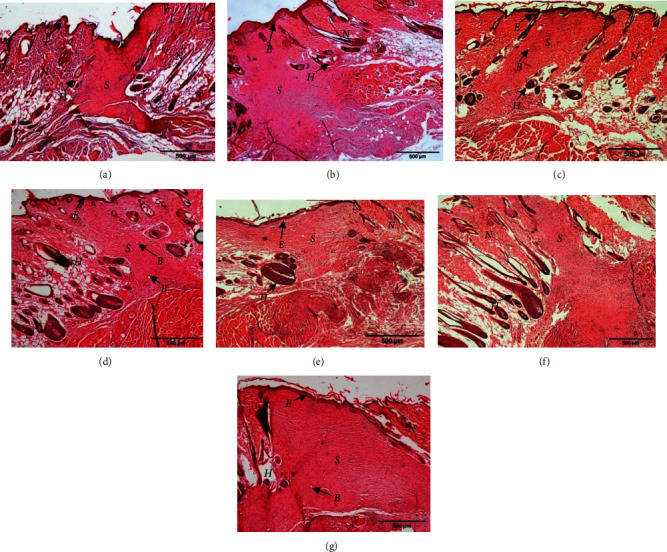
Hematoxylin & Eosin (H & E) staining of granulation healing tissue of Sprague Dawley rats treated with different treatment groups on day 14 after wounding. Seven groups were tested which were (a) untreated group, polychaete ointment (PO) with concentration of (b) 0.1%, (c) 0.5%, and (d) 1.0%, (e) cetomacrogol emulsifying ointment (negative control), (f) gamat 15%, and (g) acriflavine 0.1%. Smaller granulation tissue with denser collagen fibres and more new capillaries formation was seen in 1.0% PO than other treatments. *E* = *epidermis*, *S* = scar/granulation tissue, *N* = normal dermis, *H* = hair follicle, *B* = blood vessel. 40*X* magnification.

**Figure 4:**
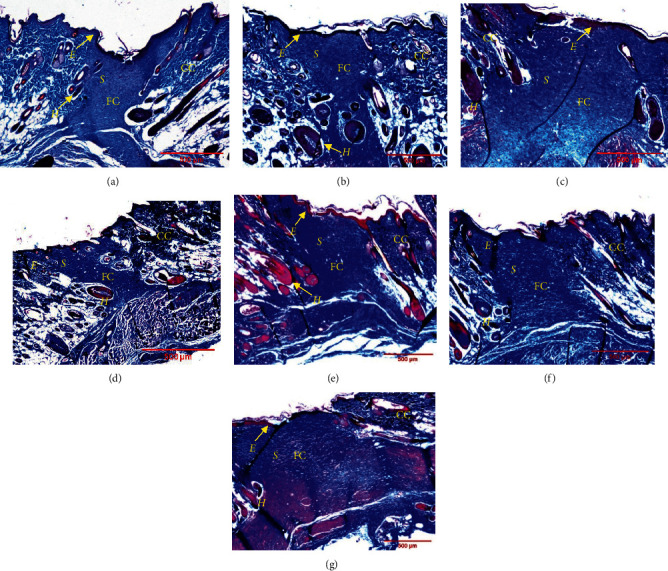
Masson's trichrome (MT) staining of granulation healing tissue of Sprague Dawley rats treated with different treatment groups at day 14 after wounding. Seven groups were tested which were (a) untreated group, polychaete ointment (PO) with concentration of (b) 0.1%, (c) 0.5%, and (d) 1.0%, (e) cetomacrogol emulsifying ointment (negative control), (f) gamat 15%, and (g) acriflavine 0.1%. New collagen formation was seen as FC and matured collagen as CC. More new collagen depositions in meshwork formation appeared in 1.0% PO. *S* = scar/granulation tissue, *E* = *epidermis*, CC = coarse collagen, FC = fine collagen, *H* = hair follicle. 40*X* magnification.

In order to evaluate the histological changes of skin in different treatment groups, wounded skin samples were collected at day 14 and stained using H & E and MT. The H & E staining revealed that all wounds had undergone the third stage of wound healing, which is proliferation phase, as they showed the appearance of new complete *epidermis* and granulation tissue (Figure 3). In detail, the result suggested that the wound treated with 1.0% of PO showed less or smaller granulation tissue with better tissue re-organisation and development of other organelles such as fibroblast fibres and hair follicles as compared to the other treatment groups. To further confirm the re-arrangement of collagen fibre in the healed tissues, MT staining was used where the collagen fibres are stained in blue colour. Moreover, using MT staining, the increase in intensity of the blue colour refers to more collagen deposition. From our finding, MT staining demonstrated denser collagen formation and being organised in meshwork arrangement in 1.0% of PO treatment group as compared to gamat treatment group (Figure 4). In detail, the intensity of the blue staining which represents fine collagen (FC) was stronger in 1.0% of PO treated group than the gamat (15%) and other treatment groups. Moreover, coarse collagen (CC) and FC were clearly seen in MT stained wounded skin. CC indicates matured collagen fibres and FC represents new collagen formation. Furthermore, thin collagen fibres were seen in MT staining of untreated, negative control, and acriflavine groups. In addition, more new blood vessels were seen in 1.0% of PO as compared to the other treatments (Figures 3 and 4).

### 3.4. Quality Assessment of *D. claparedii* Aqueous Extract Emulsifying Ointment

In this study, to ensure the quality of the PO, skin irritation, microbial contamination, and heavy metal test were assessed. This is the first study to determine the skin irritation risk of aqueous extract emulsifying ointment of *D. claparedii*. The results showed that all the tested concentrations of ointment were devoid of any signs of skin irritation such as swelling (oedema) and redness (erythema) (Table 2). The total irritation score for all concentration ointments was zero which means there was no skin reaction after three days of observation. Thus, from this result, it seems that this PO is safe to be used in wound treatment with no skin irritation.

Other than skin irritation as a potential side effect, microbial contamination is the crucial part in wound healing formulation. To assess the quality of PO, microbial contamination test was done for the best concentration of PO, i.e., 1.0%, where it showed the outstanding wound healing potential in this *in vivo* study. The result demonstrated that the values of TAMC and TYMC were not detected, indicating the number of the bacteria was less than 10^1^ CFU/g. The values for TAMC and TYMC set by British Pharmacopoeia are 10^2^ CFU/g and 10^1^ CFU/g, respectively. Furthermore, PO also did not show growth of *P. aeruginosa* and *S. aureus* (Table 3). The result suggested that the tested ointment successfully passed the criteria for microbiological quality of non-sterile dosage forms set by the British Pharmacopoeia. Finally, it showed that the aqueous extract emulsifying ointment of *D. claparedii* is safe to be used as a topical wound healing agent.

Besides, the composition of arsenic, cadmium, lead, and mercury was measured to analyse the quality of PO. Results from this study showed the levels of arsenic, cadmium, and lead were not detected, which means most of the heavy metal compositions were less than 0.1 mg/kg, except for mercury (less than 0.01 mg/kg) (Table 4). Thus, it suggested that PO is safe to be used as the level of tested heavy metals did not exceed the maximum limit set by Malaysia's National Pharmaceutical Regulatory Agency (NPRA) guideline.

### 3.5. Evaluation of Antibacterial Activity

For the present study, MIC and MBC tests were done to evaluate the antibacterial activity of *D. claparedii* aqueous extract. The result showed that aqueous extract of *D. claparedii* had weak antibacterial activity against all the five bacteria. *E. coli* and *P. aeruginosa* both showed MIC value at 0.4 g/ml (Table 5). However, MBC value for both tested bacteria is more than 0.4 g/ml because 0.4 g/ml of extract did show growth of bacteria on MHA plate. Thus, the concentration of 0.4 g/ml of extract is not sufficient to cause bactericidal effect on the bacteria. Besides, the value of MIC and MBC for *S. aureus, S. epidermidis*, and *K. pneumoniae* was more than 0.4 g/ml, as the prepared stock solution of extract (0.4 g/ml) did not show any inhibition on the tested bacteria.

### 3.6. ^1^H NMR Metabolites Identification

**Figure 5:**
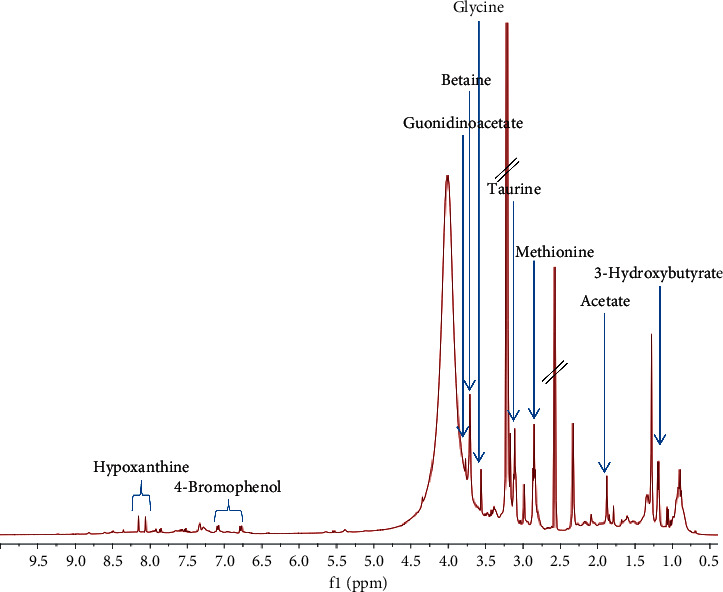
^1^H NMR spectrum of the aqueous extract of *D. claparedii* in DMSO-d_6_ containing 0.03% tetramethylsilane TMS.

Analysis of ^1^H NMR spectrum of the aqueous extract of *D. claparedii* (Figure 5) showed the presence of different classes of metabolites such as amino acids, halogenated aromatics, organic acids, vitamin, and others. The identification of metabolites was conducted based on their ^1^H NMR characteristics and comparison with the literature reports [13] and online database (https://hmdb.ca/). The detailed ^1^H NMR characteristics of the identified metabolites are presented in Table 6.

There were several amino acids that were managed to be identified in this study such as betaine, glycine, histidine, methionine, taurine, and tyrosine (Table 6). Aside from amino acids group, halogenated aromatic group such as 2-bromophenol, 4-bromophenol, and 2,4,6-tribromophenol was found in *D. claparedii* extract. Organic acids, for example, 3-hydroxyisovalerate, 3-hydroxybutyrate, acetate, and lactate, were also identified. Besides, trigonelline, a vitamin B_3_ derivative, was also present in the extract. Other metabolites that were identified include choline, creatinine, guanidinoacetate, hypoxanthine, trimethylamine, and trimethylamine N-oxide. However, there were a few prominent peaks in the spectrum that were unable to be identified due to the lack of literature and online databases.

## 4. Discussion

The capacity to regenerate segments of the body is common within phylum Annelida including polychaetes. According to a previous study, Polycheata has remarkable wound healing ability in order to regenerate their body completely. Wound healing and formation of blastemal are the earliest step in regeneration process [24]. Thus, *D. claparedii* with ability to regenerate anterior and posterior segment highly suggests that it possesses superior wound healing properties and perhaps can be used as wound healing agent. In this study, 8 mm diameter of full layer skin wound was treated with different modalities including PO from *D. claparedii* aqueous extract, commercialised gamat oil, and acriflavine. From the obtained results, PO showed obvious effect in promoting wound healing in terms of WHP and histology observation as well as having a soothing effect.

Wound healing process involved four phases of wound healing activity, which are haemostasis, inflammatory, proliferative, and remodelling phase [1, 3]. The phases were observed throughout the study and all wounds healed according to the wound healing phases. Haemostasis phase is an initial phase that aims to cease the bleeding at injury sites and it happens within seconds to minutes. Similar results were shown in this study. Bleeding happens due to microvascular injury and as a result, it is detected by a neuronal reflex mechanism that causes vasoconstriction [2]. Activation of platelets aggregation causes degranulation as well as releasing chemokines and growth factor to form a clot in order to stop the bleeding [25]. Next, inflammatory phase takes place in order to prevent infection and clear any debris as well as pathogens that invade wound site. Neutrophils are the first responders that infiltrate injury site and responsible for clearing debris and killing bacteria [3]. Neutrophils usually will remain for the first 48 hours of injury. In this study, on day 3 of observation, all wound surfaces were fresh with the presence of blood clot and decreased presence of oedema surrounding the wound as inflammation was settling down. The signs of inflammation usually are oedema, erythema, and warmth (heat) and usually associate with pain [26]. After that, proliferation phase takes place in which several activities happen together such as formation of new blood vessels (angiogenesis), granulation tissue, collagen deposition, epithelialisation, and wound contraction [1]. From the obtained result, complete re-epithelialisation was seen in almost all groups except for untreated and acriflavine group on day 11. Complete epithelialisation is defined as sloughing of the scar to leave no raw wound. It is suggested that moist environment of wound is better for re-epithelialisation process especially by using an ointment or proper dressing [4]. The trapped moisture can stimulate keratinocytes proliferation and migration as well as fibroblast growth [27].

Wound contraction is an important indicator of wound healing and usually initiated within two or three days after injury and will last for about two weeks. Cell bodies of actin and myosin of myofibroblasts are pulled together in order to close the wound [1, 3]. In the present study, 1.0% of PO showed the highest WHP throughout the experimental periods compared to other groups including positive control. However, there is no significant difference in WHP between 1.0% PO and 15% gamat oil throughout the treatment course. This explained that aqueous extract of *D. claparedii* at 1.0% concentration is comparable to the 15% concentration of commercial gamat oil. But, commercial gamat oil contains other active ingredients such as *Cocos nucifera* oil, cortex *Vitex pubescens*, and *Eucalyptus* oil, in addition to gamat extract. As expected, all of these ingredients have a positive effect on wound healing activity. Contrarily, our sample only contains *D. claparedii* crude extract and the blank ointment as a vehicle. It is also supported by a previous study that 0.5% and 1.0% aqueous extract of *Stichopus chloronotus* (one species of gamat) emulsifying ointment (with no other additive ingredients as above) showed slower wound healing effect, which were 75.0% and 60.0% of WHP, respectively, on day 10 as compared to 98.9 ± 0.3% WHP of 1.0% PO on day 11 [7]. Thus, it is suggested that polychaete has high potential as wound healing agent. The *D. claparedii* extract also showed better effects in wound healing when compared to another study using earthworm extract (100% v/v). WHP displayed by the earthworm extract was 88.3 ± 2.9% on day 11, whereas 1.0% of PO showed 98.9 ± 0.3% for the same period [16]. Altogether, these results suggested that 1.0% of *D. claparedii* ointment showed faster wound healing rate as compared to gamat and earthworm extract.

In addition, wound treated with 1.0% of PO showed less granulation tissue, more formation of new blood supply, and more collagen deposition compared to other treatments. Granulation tissue consists of fibroblasts, collagen, fibronectin, hyaluronic acid, and proteoglycan [1, 3]. Meanwhile, collagen is important in wound healing as it gives strength and integrity to the healed wound. In addition, collagen deposition, either fine or matured collagen, in wounded tissues greatly determines the phase of proliferation and remodelling phase [19]. In mammals, about 30% of total body protein is made up of collagen and it is the most abundant protein in the body. It mainly consists of amino acids that cross-link together to form a triple helix of the collagen fibril. The usual arrangements of essential amino acids are made up of glycine, proline, hydroxyproline, and arginine [1]. In addition, new blood vessels are essential to supply nutrient, oxygen, and other important necessary nutrients to wound for rapid wound healing. Neovascularisation was stimulated from angiogenic factors such as fibroblast growth factor (FGF), vascular endothelial growth factor (VEGF), platelet-derived growth factor (PDGF), angiogenin, and transforming growth factor-*α* and -ß [1]. Thus, the results suggested that 1.0% of PO has high potential as an alternative wound promoter.

The promising effect of PO on wound healing in this study could be due to some metabolites that were identified via ^1^H NMR analysis. ^1^H NMR spectroscopy is one of the analytical tools often used for the identification of metabolites in the mixture (e.g., plant extracts and biofluids) [28]. It is a rapid and simple sample preparation technique that provides comprehensive analysis of the molecular structure. In this study, some metabolites were managed to be identified in the extract such as amino acids, halogenated aromatics, organic acids, vitamins, and others. Perhaps, they are involved in wound healing mechanism of *D. claparedii*. Meanwhile, several amino acids were managed to be identified in the extract such as betaine, glycine, histidine, methionine, taurine, and tyrosine (Table 6). *Glycine*, histidine, and methionine were also present in another species of polychaete which is *Sabella spallanzanii* [29]. Amino acids are building blocks of protein and they have important role in wound healing mechanism. If there is deficiency in protein, it can cause disruption of new blood vessels formation, fibroblast proliferation, synthesis of collagen, and proteoglycan as well as wound remodelling process [30]. Studies suggested that the use of glycine in treating oral mucositis can increase cellular wound healing and collagen synthesis as well as collagen remodelling [31]. Besides, taurine or 2-aminoethanesulfonic acid is an essential amino acid that plays an important role in osmoregulation and membrane stabilisation and also has antioxidant and anti-inflammatory activities [32]. The role of antioxidant is very crucial in wound healing because it can protect cells and tissues from the action of reactive oxygen species (ROS) that can cause tissue damage. Interestingly, Norwegian red sea cucumber, *Parastichopus tremulus*, was also reported to contain high levels of taurine and glycine that are beneficial to wound healing [33]. The finding suggests these compounds may play a major role in the wound healing activity.

Halogenated aromatic groups, mainly 2-bromophenol, 4-bromophenol, and 2,4,6-tribromophenol, were identified in the spectrum. These groups were also previously isolated in polychaete of *Diopatra* genus including *D. cuprea* and *D. dentate* [13]. Bromophenols are common marine metabolites and have many pharmacological activities that may help in wound healing process such as antioxidant and antibacterial [34]. Besides, trigonelline is pyridine alkaloid and vitamin B_3_ derivative was also present in the extract. This compound is believed to have antioxidant and antimicrobial properties that can have a positive impact in the wound healing process [35]. There are other chemicals that were unable to be identified due to limited literatures. More studies need to be conducted in the future to identify these unknown chemicals. Perhaps, these compounds may play a significant role in wound healing activity too.

Besides, antibacterial properties are an added value in wound healing agent. Antibacterial agent that presents in a certain wound healing treatment can treat wound infection problem [36]. A wound will provide a suitable environment for bacteria colonisation originating from exogenous or normal skin microflora. This is due to moist, warm, and nutritious environment upon skin injury [36]. Later, a bacterial infection will impede the process of wound healing. In this study, five bacteria were chosen to test against the aqueous extract of *D. claparedii,* which were *S. aureus, S. epidermidis, E. coli*, *P. aeruginosa,* and *K. pneumoniae*. All these bacteria were commonly isolated in skin wounds. According to a previous study, 210 bacterial pathogens were isolated from 168 wound samples. From the results, *S. epidermidis* was the most isolated bacteria with 23.4% of occurrence, followed by *S. aureus* (12.5%), *P. aeruginosa* (10.9%), *E. coli* (4.7%), and *K. pneumoniae* (3.1%) [37]. In the present study, even though the extract exhibited low antibacterial activity, 1.0% of PO showed the highest WHP and more collagen deposited as compared to commercial antiseptic, acriflavine. Thus, it suggested that 1.0% PO has a great wound healing potential perhaps due to presence of bioactive compounds in the extract despite exhibiting low antibacterial activity. In this study, the results demonstrated that aqueous extract of *D. claparedii* has weak antimicrobial activity against Gram-negative and Gram-positive bacteria. A previous study demonstrated that different types of solvent extraction (water, methanol, and acetone) of polychaete, *Perinereis cultrifera*, showed antimicrobial activities towards 10 different bacteria [38]. From the study, *S. aureus* and *E. coli* were highly inhibited by methanol extract compared to water and acetone. Meanwhile, *P. aeruginosa* was not inhibited by all three extracts. Thus, it suggested that perhaps more polar compounds in the polychaete extract exhibit low antibacterial activity compared to less polar compounds. Furthermore, absence of or low antibacterial activity does not depict the absence of bioactive compounds in the test subject. But, the results may be due to inadequate active constituents in the extract to exhibit better antimicrobial activity [39].

Apart from positive potential effect on wound healing, 1.0% PO also is safe to use as topical wound healing treatment. The quality assessments of 1.0% PO were done that include skin irritation, microbial contamination, and heavy metal test. Skin irritation is one of the side effects of drugs, which is commonly reported in synthetic drugs. It is defined as reversible damage that is caused from the test substance [20]. The test is important to establish scientific data regarding the potential risk of dermal irritation for new substances or products that will be utilised by consumers [40]. This is the first study to determine the skin irritation risk of aqueous extract emulsifying ointment of *D. claparedii* and no signs of skin irritation were detected. Oedema and erythema are usual signs of allergic response. Erythema or redness of the skin is caused by hyperaemia or increase blood flow in superficial capillaries. Meanwhile, oedema is swelling due to the increase of interstitial fluid. They are caused due to stimulus from allergens that trigger immune response to release proinflammatory cytokines such as histamine and leukotrienes [40]. In this study, utilisation of water as an extraction solvent for *D. claparedii* reduces the chances of contribution to skin irritation. Generally, water is used as an extraction solvent in traditional medicine since it is safe, eco-friendly, and cost-effective [41]. In addition, no chemical was added in the preparation of PO. Cetomacrogol emulsifying ointment was used as a vehicle in preparation of PO. No skin irritation was observed in cetomacrogol as well. This emulsifying ointment is mainly composed of liquid paraffin, white soft paraffin, and cetomacrogol emulsifying wax. It is slightly greasy, glossy, and not water-washable, so that it can remain longer on the skin and keep delivering the medicinal effects to the target site [42]. Thus, from this result it seems that this PO is safe to be used in wound treatment with no skin irritation.

Besides, microbial contamination test for non-sterile dosage forms is important to assess the safety of products for human consumption [43]. In this study, results suggested that PO was safe to use as no microbial contamination was detected. TAMC and TYMC reflect general contamination and the presence of a favourable environment for the growth of microorganisms [43]. The absence of *S. aureus* and *P. aeruginosa* ensures that the used aqueous extract emulsifying ointment of *D. claparedii* will not contaminate and colonise the wounds since these bacteria were commonly isolated from the wounded skin [37].

In addition, heavy metals are naturally found in the environment; hence, they exist in raw materials used in the processing of products including cosmetic and healthcare [44]. In Malaysia, NPRA as a drug control authority is responsible for ensuring that pharmaceutical substances, traditional medicines, and cosmetic products are safe to be used by consumers. Limit test for heavy metals is one of the analyses for product safety. In this study, the level of tested heavy metals in PO did not exceed the maximum limit set up by NPRA. Thus, PO is safe to use as a topical wound healing agent. Moreover, it is essential to measure the composition of heavy metals because they can cause many complications to human health at higher concentrations. For instance, lead is a neurotoxin that can impair language, learning, and behaviour ability. Meanwhile, mercury is hazardous due to toxicity to the nervous, reproductive, immune, and respiratory system [44].

## 5. Conclusions

In this study, the wound healing potential of the marine worm, *D. claparedii*, in ointment form was revealed for the first time using Sprague Dawley rats. The results demonstrated that, among the other treatment groups, 1.0% (w/w) of *D. claparedii* ointment is the most effective in wound healing process in terms of soothing effect, faster wound healing rate, more collagen deposition, and less scar. This might be due to the involvement of different classes of metabolites found in the polychaete extract that positively impact the wound healing process. For instance, amino acids, halogenated aromatics, organic acids, vitamins, and other chemicals were unable to be identified due to limited literatures. More studies need to be conducted in the future to identify these unknown chemicals. However, the aqueous extract of *D. claparedii* exhibited low antibacterial activities against *E. coli* and *P. aeruginosa*. It may contribute to the presence of polar compounds with low antibacterial activity found in the extract. Additionally, the proposed ointment is safe to be applied on skin with no local skin irritation, microbial presence, and insignificant concentration of heavy metals. Overall, it is suggested that *D. claparedii* could be utilised as a promising and alternative wound healing agent with minimal side effects in the future.

## Figures and Tables

**Table 1:** Three categories of behaviours of Sprague Dawley rats were observed on days 0, 3, 7, 11, and 14 of after wounding. Three criteria listed are withdrawal pain response (wound licking/scratching), sleeping/resting, and cage exploration (walking/climbing). All rats showed similar pattern of behaviour throughout healing process. Seven treatment groups were tested which were 0.1%, 0.5%, and 1.0% of polychaete ointment (PO), gamat (15.0%), acriflavine (0.1%), cetomacrogol emulsifying ointment (negative control), and untreated group.

Treatment	Day/Activity		0	3	7	11	14
Untreated	Wound licking/scratching		+				
	Sleeping/resting			+	+		
	Cage exploration	Walking			+	+	+
		Climbing				+	+

0.1% PO	Wound licking/scratching		+				
	Sleeping/resting			+	+		
	Cage exploration	Walking			+	+	+
		Climbing				+	+

0.5% PO	Wound licking/scratching		+				
	Sleeping/resting			+	+		
	Cage exploration	Walking			+	+	+
		Climbing				+	+

1.0% PO	Wound licking/scratching		+				
	Sleeping/resting			+	+		
	Cage exploration	Walking			+	+	+
		Climbing				+	+

Negative control	Wound licking/scratching		+				
	Sleeping/resting			+	+		
	Cage exploration	Walking			+	+	+
		Climbing				+	+

Gamat (15.0%)	Wound licking/scratching		+				
	Sleeping/resting			+	+		
	Cage exploration	Walking			+	+	+
		Climbing				+	+

Acriflavine (0.1%)	Wound licking/scratching		+				
	Sleeping/resting			+	+		
	Cage exploration	Walking			+	+	+
		Climbing				+	+

+ indicates presence of behaviour activity.

**Table 2:** Skin irritation score after different concentrations of polychaete ointment (PO) applied on intact skin over period of time. Concentrations of PO were 0.1%, 0.5%, and 1.0%. Cetomacrogol emulsifying ointment was used as negative control. No signs of skin irritation (oedema and erythema) were observed for all the tested ointments.

Skin reaction	Observation time (hr)	Treatment				Total score
		Negative control	0.1% PO	0.5% PO	1.0% PO	
Erythema formation	1	0	0	0	0	0
	24	0	0	0	0	0
	48	0	0	0	0	0
	72	0	0	0	0	0

Oedema formation	1	0	0	0	0	0
	24	0	0	0	0	0
	48	0	0	0	0	0
	72	0	0	0	0	0

**Table 3:** Microbial contamination test result for 1.0% of aqueous extract emulsifying ointment of *D. claparedii*. Results successfully passed the criteria for microbiological quality of nonsterile dosage forms set by British Pharmacopoeia.

Test description	Unit	Result	Acceptance criteria
Total aerobic microbial count (TAMC)	cfu/g	ND < 10	10^2^
Total yeast and mould count (TYMC)	cfu/g	ND < 10	10^1^
*Pseudomonas aeruginosa*		Absent in 0.1 g	Absent in 1 g
*Staphylococcus aureus*		Absent in 0.1 g	Absent in 1 g

ND = not detected.

**Table 4:** Heavy metal test result for 1.0% of aqueous extract emulsifying ointment of *D. claparedii*. All tested heavy metals did not exceed National Pharmaceutical Regulatory Agency (NPRA) limit.

Heavy metal	Unit	Result	NPRA limit
Arsenic	mg/kg	ND < 0.1	5
Cadmium	mg/kg	ND < 0.1	5
Lead	mg/kg	ND < 0.1	20
Mercury	mg/kg	ND < 0.01	1

ND = not detected.

**Table 5:** Value of MIC, MBC, and MIC-to-MBC ratio of *D. claparedii* aqueous extract against five selected bacteria. MIC value for *E. coli* and *P. aeruginosa* was 0.4 g/ml and not detected for *S. aureus*, *S. epidermidis*, and *K. pneumoniae*.

Bacteria	MIC value (g/ml)	MBC value (g/ml)	MBC : MIC ratio
*S. aureus*	>0.4	>0.4	ND
*S. epidermidis*	>0.4	>0.4	ND
*E. coli*	0.4	>0.4	>1
*P. aeruginosa*	0.4	>0.4	>1
*K. pneumoniae*	>0.4	>0.4	ND

Note. ND = not detected.

**Table 6:** Tentative metabolites identified in *D. claparedii* aqueous extract using ^1^H NMR.

Metabolite	Chemical shift (*δ* ppm)
*Amino acids*	
Betaine	3.16 (*s*), 3.76 (*s*)
Glycine	3.55 (*s*)
Histidine	3.08 (*m*), 3.29 (*m*), 3.98 (*m*), 7.11 (*s*), 7.19 (br *s*)
Methionine	2.02 (*m*), 2.08 (*s*), 2.17 (*m*), 2.66 (*t*, *J* = 8.0 Hz), 3.89 (*m*)
Taurine	2.83 (*t*, *J* = 6.4 Hz), 3.10 (*t*, *J* = 6.4 Hz)
Tyrosine	3.03 (*m*), 3.19 (*m*), 3.94 (*m*), 6.86 (*m*), 7.19 (*m*)

*Halogenated aromatics*	
2-Bromophenol	6.98 (*d*, *J* = 6.8 Hz), 7.86 (*d*, *J* = 7.2 Hz)
4-Bromophenol	6.79 (*d*, *J* = 8.4 Hz), 7.10 (*d*, *J* = 8.4 Hz)
2,4,6-tribromophenol	7.77 (*s*)

*Organic acids*	
3-Hydroxyisovalerate	1.27 (*s*), 2.32 (*s*)
3-Hydroxybutyrate	1.17 (*d*, *J* = 6.4 Hz), 2.15 (*m*), 2.27 (*m*), 4.14 (*m*)
Acetate	1.87 (*s*)
Lactate	1.33 (*d*, *J* = 8 Hz), 4.13 (*m*)

*Vitamin*	
Trigonelline	4.41 (*s*), 8.09 (*m*), 8.96 (*m*), 9.22 (*m*)

*Others*	
Choline	3.16 (*s*), 3.48 (*m*), 3.92 (*m*)
Creatinine	3.06 (*s*), 4.05 (*s*)
Guanidinoacetate	3.76 (*s*)
Hypoxanthine	8.04 (*s*), 8.14 (*s*)
Trimethylamine	2.97 (*s*)
Trimethylamine N-Oxide	3.28 (*s*)

Note. The type of peaks is listed for each metabolite, where *s* = singlet, br *s* = broad singlet, *d* = doublet, *t* = triplet and *m* = multiplet. The coupling constant, *J*, is a measure of the interaction between a pair of protons.

## Data Availability

The data used to support the findings in this study are included within the article.
